# Peptidoglycan enzymes of *Francisella*: Roles in cell morphology and pathogenesis, and potential as therapeutic targets

**DOI:** 10.3389/fmicb.2022.1099312

**Published:** 2023-01-12

**Authors:** Beth A. Bachert, Joel A. Bozue

**Affiliations:** Bacteriology Division, The United States Army Medical Research Institute of Infectious Diseases (USAMRIID), Frederick, MD, United States

**Keywords:** *Francisella*, peptidoglycan, tularemia, antibiotics, therapeutics, lytic transglycosylase, carboxypeptidase, penicillin-binding protein

## Abstract

Peptidoglycan, found within the cell wall of bacteria, is a structure critical for maintaining cell morphology and providing a protective barrier in diverse environments. Peptidoglycan is a remarkably dynamic structure that is constantly remodeled during cell growth and division by various peptidoglycan enzymes. Numerous peptidoglycan enzymes have been characterized from diverse bacteria and are highly sought after as targets for therapeutics. However, very little is known about these enzymes within the biothreat agent *Francisella tularensis*. As the causative agent of tularemia, *F. tularensis* is classified as a category A biothreat pathogen, in part due to its low infectious dose and lack of FDA-approved vaccine. Many bacterial species encode multiple peptidoglycan enzymes with redundant functions that allow for compensation if one of the enzymes are inactivated. In contrast, *F. tularensis* appears to lack this redundancy, indicating peptidoglycan enzymes may be completely essential for growth and could be exploited as targets for medical countermeasures. Indeed, several peptidoglycan enzymes in *F. tularensis* have been shown to play important roles in cell division, cell morphology, virulence, and modulation of host response. The aim of this review is to summarize findings from the current literature on peptidoglycan enzymes present in *Francisella* and discuss areas where future research efforts might be directed. We conclude that *Francisella* harbors a distinct set of peptidoglycan enzymes important for cell growth and virulence and represent potentially valuable targets for the development of novel therapeutics.

## Introduction

The vast majority of bacteria contain a cell wall consisting of peptidoglycan (PG), a structure critical for maintaining the cell morphology and membrane integrity, and providing a protective barrier against osmotic pressure, antibacterial compounds, and other environmental stresses. PG is composed of alternating *N*-acetylglucosamine (Glc*N*Ac) and *N*-acetylmuramic acid (Mur*N*Ac) residues connected by β-(1–4) linkages. These glycan strands are further crosslinked by peptide chains, a feature which contributes to the cell wall stability and resistance to changes in osmotic pressure. In Gram-negative bacteria, these peptide chains are typically composed of L-Ala-γ-D-Glu-diaminopimelate (meso-DAP)-D-ala-D-ala, with the L-ala residue attaching the peptide chain to the C3 position of the Mur*N*ac residue (Vollmer et al., 2008; Sobhanifar et al., 2013). While these features are characteristic of most Gram-negative bacteria, some level of variation in the glycan strands or peptide chains exists across bacterial species (Schleifer and Kandler, 1972). Glycan strands are then further linked by short peptide chains to form a thin meshwork that surrounds the inner membrane. The number of peptidoglycan layers has been estimated to be between 1 and 3 in *Escherichia coli*, depending on the strain and growth conditions (Yao et al., 1999; Matias et al., 2003).

The bacterial cell wall is a dynamic structure that undergoes constant remodeling to accommodate cell division, response to environmental signals, and incorporation of membrane proteins. While the PG layer offers structural stability to the cell wall, it must also be flexible to accomplish these tasks. PG remodeling relies on the coordinated activities of a series of modifying enzymes, which target the glycan strands and the peptide cross-links. Extensive knowledge has been obtained from studying the array of PG remodeling proteins in *E. coli* and other bacteria. However, very few studies have investigated the PG remodeling enzymes in *Francisella* spp., and no characterization of the PG structure or composition in this organism has been performed. *Francisella tularensis*, the causative agent of tularemia, is a Gram-negative facultative intracellular bacterium. Within *F. tularensis*, there are two subspecies that cause disease in humans, subsp. *tularensis* and subsp. *holarctica*. A third subspecies, *F. novicida*, is commonly used as a laboratory surrogate for the fully virulent strains*. F. tularensis* poses a significant biothreat risk due its low infectious dose, ease of aerosolization, and lack of approved vaccine (McLendon et al., 2006). Moreover, the potential for antibiotic resistant strains makes finding novel antibiotics and therapeutics critical (Gestin et al., 2010; Sutera et al., 2014; Jaing et al., 2016; Biot et al., 2020). As a facultative intracellular pathogen, *F. tularensis* is able to replicate within multiple host cells, primarily macrophages, which is necessary for pathogenesis. Once taken up by a macrophage, *F. tularensis* resides within a phagosome. The phagosomal membrane is then disrupted, and the bacterium released to replicate freely within the cytosol. More detailed reviews on the intracellular life cycle of *F. tularensis* are available (Clemens and Horwitz, 2007; Chong and Celli, 2010; Santic et al., 2010). Exploring the PG enzymes of *Francisella* is an exciting area of study, given the importance of PG enzymes in cell division, virulence, antibiotic resistance, and their potential as therapeutic targets. The focus of this review is to summarize the current knowledge of PG enzymes in *Francisella* and highlight gaps in our understanding that require further investigation. We hope this work becomes part of the larger effort to reveal potential targets to combat tularemia.

### Overview of peptidoglycan enzymes

The initial steps of PG biosynthesis occur in the cytosol *via* the Mur pathway that results in the formation of the lipid II PG precursor, consisting of a single disaccharide, pentapeptide unit attached to the lipid carrier (Egan et al., 2020). This precursor is then flipped across the inner membrane to allow for subsequent polymerization of the PG, which is carried out primarily by penicillin-binding proteins (PBPs). PBPs are a large group of enzymes broadly divided into two classes, high molecular mass (HMM) and low molecular mass (LMM) PBPs (Sauvage et al., 2008). The HMM PBPs are involved in the first steps of PG synthesis in the periplasm, catalyzing glycosyltransferase and transpeptidase reactions, involving transfer of the growing PG chain to the PG precursor and cross-linking of the glycan chains, respectively. The LMM PBPs are involved in cell separation and PG recycling (Sauvage et al., 2008). For the purpose of simplicity and therapeutic applications of the PG enzymes, this review will focus on the final steps of PG synthesis and recycling that occur in the periplasmic space.

**Figure 1:**
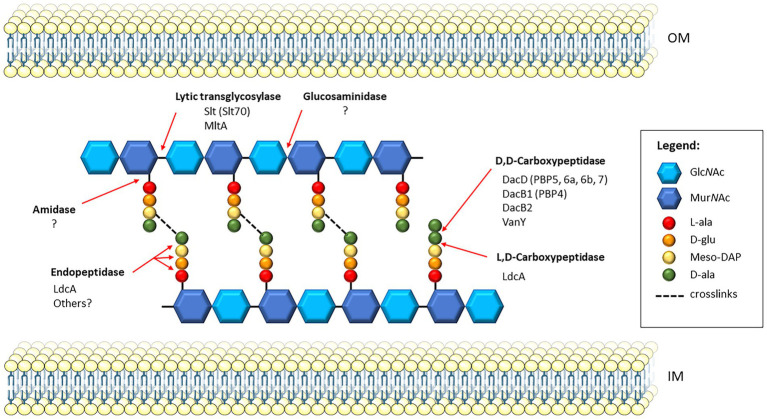
Schematic of PG and PG recycling enzymes in *Francisella*. A section of cross-linked PG is shown between the inner membrane (IM) and outer membrane (OM) of the bacterial cell. Since *Francisella* PG has not yet been characterized, this model shows the predicted PG composition based on that of *Escherichia coli*. The glycan strands composed of alternating disaccharide residues, *N*-acetylglucosamine (Glc*N*Ac) and *N*-acetylmuramic acid (Mur*N*Ac), are crosslinked *via* the stem peptide. The stem peptide is composed of L-Ala-γ-D-Glu-diaminopimelate (meso-DAP)-D-ala-D-ala. Transpeptidases catalyze the formation of crosslinks in a two-step process, first removing the terminal D-ala of the stem peptide, then linking the D-ala of the resulting tetrapeptide on donor strand to the meso-DAP residue on the acceptor strand. Red arrows indicate the cleavage sites of specific PG enzymes, and those known to be present in *Francisella*; the homologous enzymes in *E. coli*, if applicable, are indicated in parentheses. *Francisella* harbors two known lytic transglycosylases, Slt and MltA, which cleave the β-1,4 glycosidic bond between Mur*N*Ac and Glc*N*Ac residues. Currently, no glucosaminidases have been identified in *Francisella*, which also act upon glycan strands but cleave the non-reducing end of the Glc*N*Ac residues. Five D, D-carboxypeptidases are present in *Francisella*, including DacD, DacB1, DacB2, and VanY. The LdcA enzyme is an L,D-carboxypeptidase that also exhibits activity as an endopeptidase, though no others have been yet identified. Lastly, amidases, which cleave the bond between the Mur*N*ac residues and the stem peptide, have not been identified in *Francisella*.

The LMM PBPs consist of both carboxypeptidases, which cleave the terminal D-alanine from the stem pentapeptide creating a tetrapeptide, and endopeptidases, which cleave within the peptide crosslinks (Aguilera Rossi et al., 2016). Cell wall hydrolases, also referred to as autolysins, consist of three enzyme types, amidases, peptidases, and glycosidases, all of which are involved in cell wall remodeling during cell division. These enzymes are distinct from PBPs, though share overlapping functions (Vermassen et al., 2019). The amidases cleave the bond between the peptide stem and glycan chain, peptidases cleave the peptide stem, and glycosidases act upon the glycan chains. The lytic transglycosylases (LTs), structurally related to the glycosidases, are a unique family of enzymes that cleave the beta-glycosidic bond between the N-acetylglucosamine and N-acetylmuramic acid residues of the glycan strands, and catalyze the formation of 1,6-anhydro-N-acetyl-β-D-muramoyl residue (Dik et al., 2017). The schematic in Figure 1 shows where in the PG each type of enzyme cleaves, along with the known PG enzymes in *Francisella* spp. Currently, there are at least five carboxypeptidases and two lytic transglycosylases known to be present in *Francisella*, with LdcA harboring both carboxypeptidase and endopeptidase activity, and no known amidases or glucosaminidases reported in the literature. Known and putative peptidoglycan enzymes and their locus tags in three representative *Francisella* strains are listed in Table 1. Both LT enzymes from *Francisella* and two of the carboxypeptidases, DacD and LdcA, have been investigated and will be discussed herein.

**Table 1:** PG enzymes and corresponding locus tags in the three *Francisella* strains of interest.

Gene	*Francisella tularensis* Schu S4	*Francisella holarctica* live vaccine strain (LVS)	*Francisella novicida* U112	Protein product
*ldcA* ^1^	FTT_0101	FTL_1678	FTN_1613	L,D-carboxypeptidase
*dacD* ^2^	FTT_1029	FTL_1060	FTN_0907	D,D-carboxypeptidase
*slt* ^2^	FTT_0400	FTL_0466	FTN_0496	Soluble lytic murein transglycosylase
*mltA* ^2^	FTT_1271	FTL_1189	FTN_1286	Membrane-bound lytic murein transglycosylase A
*dacB1* ^3^	FTT_1039	FTL_1046	FTN_0917	D,D-carboxypeptidase
*dacB2* ^3^	FTT_0724c	FTL_1509–1,508	FTN_0635	D,D-carboxypeptidase
*vanY* ^3^	FTT_0549	FTL_1004	FTN_0966	D,D-carboxypeptidase

^1^Enzymatic activity has been demonstrated (Zellner et al., 2021). ^2^Described and characterized, discussed herein, but the specific enzymatic activity not shown. ^3^Putative PG enzymes based on homology.

### Carboxypeptidases

The D-alanyl-D-alanine carboxypeptidases, or DD-carboxypeptidases, are the most abundant PBPs within *E. coli*, though mutation of one or more genes does not cause lethality, owing to the highly redundant nature of these enzymes (Denome et al., 1999). The major DD-carboxypeptidase in *Francisella*, DacD, has been characterized in two separate studies. In the first study, *dacD* of the *F. holarctica* strain FSC200 was inactivated *via* insertional mutagenesis and shown to have a significant replication defect in murine bone-marrow derived macrophages (Spidlova et al., 2018). The *dacD* mutant was also attenuated in BALB/c mice after subcutaneous infection, with decreased spread to major organs in comparison to the wild-type strain. Transmission electron microscopy (TEM) revealed significantly increased cell size and breaks within the plasma membrane of the *dacD* mutant. The latter study generated an insertional *dacD* mutant in the fully virulent *F. tularensis* Schu S4 strain and showed a pH-dependent growth defect of the mutant relative to wild-type, that was restored by complementation and partially restored for growth at neutral pH (Kijek et al., 2019). Moreover, the *dacD* mutant had significant morphological defects and increased cell size as evidenced by scanning electron microscopy (SEM). Finally, during intranasal infection of BALB/c mice, the *dacD* mutant was significantly attenuated for virulence and protective against challenge with the parent Schu S4 strain, showing its potential as a live vaccine strain.

Recently, the L, D-carboxypeptidase, LdcA, of *F. tularensis* was described, which cleaves pentapeptide stems to tripeptide stems, as opposed to the D, D-carboxypeptidases which cleave the terminal D-alanine to form tetrapeptide stems (Zellner et al., 2021). Using a recombinant LdcA protein derived from the live vaccine strain (LVS), the authors demonstrated both L, D-carboxypeptidase and L, D-endopeptidase activities that were dependent on at least two residues of the Ser-Glu-His catalytic triad. Isogenic deletion of *ldcA* in LVS produced cells that were smaller and rounder than wild-type cells, with significantly thicker outer membranes and septum defects indicated by the presence of bacterial chains. The virulence of this mutant was completely attenuated during intranasal infection of C3H/HeN mice as compared to wild-type and complement strains, and further protected against challenge with the fully virulent Schu S4 strain.

Additional DD-carboxypeptidases, including DacB1 (FTL_1046), DacB2 (FTL_1509) and VanY (FTL_1004), have been annotated in *Francisella* spp. (Table 1), though no studies have yet been published on the characterization and confirmation of these putative enzymes. It is possible that additional PG enzymes are present in *Francisella* though not annotated; indeed, lack of consistent sequence annotation is a challenge when analyzing available genome data for *Francisella*. Homologs may be identified *via* BLAST search of known PG enzyme sequences, although *Francisella* harbors many hypothetical proteins and proteins which bear no homology to existing proteins, even if they exhibit similar functions. Interestingly, a previous study identified FTT_0924 as an essential gene in *F. tularensis*, encoding an unknown but highly conserved protein that was necessary for maintaining PG stability and replication in macrophages. This protein could represent one of the PG enzymes employed by *Francisella* spp., to maintain proper cell division (Brunton et al., 2015). Roughly 30% of the genes identified in *Francisella* are hypothetical proteins of unknown function (Titball and Petrosino, 2007).

### Lytic transglycosylases

*Francisella tularensis* harbors only two known lytic transglycosylases, the soluble lytic transglycosylase (Slt), and the murein lytic transglycosylase A (MltA). The role of the Slt enzyme in cell morphology and virulence was previously examined using a transposon mutant of *F. novicida* (Bachert et al., 2019). The *slt* mutant showed significant growth defects and loss of viability during growth in low pH conditions. SEM further showed morphological defects including aberrant cell shapes and sizes, and extensive clumping and fusion of cells which were restored *via* complementation. The *F. novicida slt* mutant was also significantly attenuated during intranasal infection of BALB/c mice, with limited dissemination to major organs, and showed decreased replication in J774 murine macrophage-like cells (Bachert et al., 2019). The *slt* gene was also identified in a cytotoxicity study, in which a transposon mutant of *slt* was found to be less cytotoxic to THP-1 human monocytes than the parental *F. novicida* strain (Nakamura et al., 2019). The authors further constructed an in-frame deletion of *slt* in *F. novicida* and showed reduced growth of the mutant in both THP-1 cells and J774 cells. Although the mutant was still able to escape the phagosome, mutant bacteria were degraded by autophagy during the later stages of infection. Furthermore, the mutant elicited high levels of pro-inflammatory cytokines TNF-α, IL-6, and IL-1β from the cells, indicating a role for Slt in immune suppression (Nakamura et al., 2019).

More recently, Nakamura et al. (2020) identified the related MltA enzyme as necessary for the survival of *F. novicida* in silkworms. Virulence in the silkworm model was significantly attenuated using an in-frame deletion mutant of *mltA* and restored by complementation. MltA was shown to suppress immune responses in this model as evidenced by increased induction of cecropin B expression in the *mltA* mutant. Lastly, *F. novicida mltA* failed to replicate in THP-1 cells and was less cytotoxic as assessed by LDH release (Nakamura et al., 2020).

## Discussion

### Lack of peptidoglycan enzymes redundancy in *Francisella*

Peptidoglycan modifying enzymes are reported to be highly redundant in a variety of bacteria. For example, *E. coli* harbors at least 35 PG hydrolases, including 7 carboxypeptidases and 8 lytic transglycosylases (van Heijenoort, 2011). *Pseudomonas aeruginosa* expresses 11 LTs (Lee et al., 2017). However, *Francisella* species appear to lack the redundancy of PG enzymes observed in many bacteria. A previous search for *E. coli* PBP homologs revealed only three PBPs in *F. tularensis*, in contrast to 12 PBPs in *E. coli*. The *F. tularensis* homologs are DacD, FtsI, and DacB1 with DacD representing the major DD-carboxypeptidase homologous to PBP5, 6a, 6b, and 7 from *E. coli* (Kijek et al., 2019). Furthermore, *F. tularensis* has been found to harbor only two LT enzymes, MltA (Nakamura et al., 2020) and Slt (Bachert et al., 2019; Nakamura et al., 2019). Moreover, *dacD*, *dacB1*, *ftsI*, *slt*, and *mltA* have all been identified as essential for *in vitro* growth of *F. tularensis* using a transposon library (Ireland et al., 2019). This finding does not rule out potential polar effects of a transposon insertion. However, multiple attempts by our laboratory to generate in-frame deletions of *slt* in LVS and the Schu S4 strain of *F. tularensis* were unsuccessful and suggest *slt* is essential for *F. tularensis* (Bachert et al., 2019). Such essential genes would be ideal targets for medical countermeasures against tularemia. The lack of gene redundancy observed is not unique to *F. tularensis*. It has been shown that host-evolved bacteria with reduced genomes, including *Mycobacterium leprae* and *Rickettsia prowazekii*, lost their genetic redundancy in favor of maintaining diverse protein families, in contrast to free living bacteria (Mendonça et al., 2011). Conversely, for pathogens with large genomes and frequent gene duplication, including duplication of PG enzymes, the effectiveness of potential PG enzymes inhibitors could be limited.

### Impact of peptidoglycan enzymes on *Francisella* cell morphology

The L, D- and D, D-carboxypeptidases of *Francisella* appear to have important but very distinct roles in cell morphology, although they both act upon the peptide cross-links of the cell wall. For instance, inactivation of *dacD* resulted in a pH-dependent growth defect, but the *ldcA* mutant showed no replication defect *in vitro* (Kijek et al., 2019; Zellner et al., 2021). Moreover, some of the morphological defects observed for LVS *ldcA* were opposite those of the *dacD* mutant, including smaller rather than larger cell size and a thicker outer membrane compared to a discontinuous membrane (Spidlova et al., 2018; Zellner et al., 2021). These mutations have complex effects on the bacterial cell surface and its sensitivity to various stressors. For instance, *ldcA* mutant had increased sensitivity to cell-wall targeted antibiotics, such as vancomycin and ampicillin, but increased resistance to cytosol-targeted antibiotics such as gentamicin and ciprofloxacin (Zellner et al., 2021). Notably, increased sensitivity to sodium dodecyl sulfate was observed for both *dacD* and *ldcA* mutants (Spidlova et al., 2018; Kijek et al., 2019; Zellner et al., 2021). Interestingly, the *ldcA* mutant was reported to form chains of cells for ~10% of the bacteria (Zellner et al., 2021). We saw a similar phenotype with the *slt* mutant, although with clumping of cells rather than chains; Slt also acts upon the glycan strands rather than the cross-links (Bachert et al., 2019).

The overall phenotypes of *slt* and *dacD* mutants were highly similar, both showing increased cell size and altered membrane structure. Both mutants also exhibited pH-dependent phenotypes, with membrane defects apparent in low pH conditions that were partially restored by growth in neutral pH conditions (Bachert et al., 2019; Kijek et al., 2019). This pH-dependency could point to a role for Slt and DacD enzymes in maintaining bacterial cell morphology in the intracellular environment of the macrophage during host infection. The *slt* and *dacD* mutants were characterized using SEM, which allowed for the visualization of surface structures and observation of ruffled surface appearance, while the *ldcA* mutant was characterized using TEM; SEM analysis of the *ldcA* mutant could potentially reveal similar defects. It is also worth noting that mutation of the genes encoding for these enzymes may have different effects depending on the strain or subspecies. For example, mutation of *dacD* in the *F. tularensis* Schu S4 strain resulted in cells roughly two times larger than wild-type (Kijek et al., 2019), whereas mutation of *dacD* in the *F. holarctica* FSC200 strain resulted in cells 10 times larger than wild-type (Spidlova et al., 2018). No morphological defects have yet been reported for *mltA* mutants of *Francisella*, although the attenuated growth and virulence of *F. novicida mltA* would suggest such a phenotype.

### Roles of peptidoglycan enzymes in immunomodulation

Interestingly, both Slt and MltA were implicated in immunomodulation, although discovered in different models of infection, i.e., THP-1 cells for Slt and silkworm for MltA (Nakamura et al., 2019, 2020). It is possible that aberrations in the cell wall resulting from mutation of the genes encoding for the LT enzymes alters the recognition of the PG by the TLR2 receptor. It would be interesting to test *Francisella mltA* and *slt* mutants in parallel using a mouse model and obtaining cytokine profiles of the host challenged with the mutants compared to wild-type infected mice. Additionally, since the LT enzymes have been associated with assembly of the type VI secretion system and incorporation into the cell envelope of *E. coli* (Santin and Cascales, 2017), the immunosuppressive effects observed with some of these mutants may be due in part to alterations in the type VI secretion system. However, the contribution of these enzymes to type VI secretion system assembly or stability in *Francisella* spp., are currently unknown.

Modulation of the immune response by PG fragments released during cell wall recycling of bacteria has been reported in the literature. This phenomenon is especially well-characterized in *Neisseria gonorrhoeae* and *Neisseria meningitidis*, where toxic PG fragments, released from the activity of LTs during host infection, contribute to inflammation and cytotoxicity (Chan and Dillard, 2017). This is in contrast to the LTs of *Francisella spp.*, which have been shown to dampen immune responses as described above. In general, *Francisella* is a stealth pathogen, replicating within macrophages without triggering host defenses or causing overt damage (Sjöstedt, 2006). The modified LPS structure expressed by *Francisella* is known to bypass typical TLR4 recognition, contributing to its ability to replicate unseen within the host (Okan and Kasper, 2013). However, the structure of the PG within *Francisella* has not yet been characterized and may harbor modifications that allow the bacterium to suppress or evade host immune responses.

NOD-like receptors within host cells are known to recognize the presence of intracellular PG in host cells and trigger the inflammasome, leading to caspase-1 activation and the production of IL-1B and IL-18 leading to cell death. Similar to other intracellular pathogens, *Francisella* has been shown to activate the inflammasome and caspase-1 responses (Weiss et al., 2007; Broz and Monack, 2011). However, the mechanisms described rely on AIM2 sensing of dsDNA and NLRP3 sensing of LPS (Man et al., 2015; Mitra et al., 2018). Whether *Francisella* PG within host cells triggers a similar immune response is not currently known.

### Implications in targeting

Targeting the lytic transglycosylases, specifically in combination with beta-lactam antibiotics, may be useful for the design of novel therapeutics. Bulgecin A, an O-sulfonated glycopeptide, is an LT enzyme inhibitor that was first demonstrated to be active against the Slt70 enzyme of *E. coli*, and the Slt70-Bulgecin A complex was crystallized to determine the structure (Thunnissen et al., 1995). While Bulgecin A harbors no antibiotic activity itself, it works synergistically with beta-lactams to increase their efficacy. The combination treatment of Bulgecin A and beta-lactams has been demonstrated effective against multiple pathogens, including *E. coli*, *P. aeruginosa*, *Helicobacter pylori*, and *Acinetobacter baumannii* (Templin et al., 1992; Thunnissen et al., 1995; Bonis et al., 2012; Skalweit and Li, 2016). Bulgecin A has not yet been tested against Francisella, either alone or in combination with beta-lactams. Bulgecin A harbors great potential for improving efficacy of existing drugs when used as a combinational therapy.

## Conclusion

Peptidoglycan enzymes have long been considered as potential targets for antibiotics, given that it is unique to bacteria and the active sites of these enzymes have been well characterized in common pathogens, such as *E. coli*. While very little is known about how PG remodeling occurs in *Francisella* and the specific enzymes involved, the few studies discussed above highlight a significant role of PG enzymes in the survival and virulence of *Francisella*. Importantly, the LT enzymes are essential for survival in fully virulent *F. tularensis* and when mutated in *F. novicida*, have a significant impact on cell morphology and virulence. This is in contrast to *E. coli* and other species, in which mutation of multiple PG enzymes result in no detected phenotype. These observations suggest antibiotics targeting the essential LT enzymes of *Francisella* may be especially potent against tularemia. Moreover, the threat of antibiotic resistance heightens the biothreat concern of *Francisella*, making the development of novel antibiotics and inhibitors essential. The active sites for LTs and carboxypeptidases seem to be conserved between *Francisella* and *E. coli*, so existing antibiotics may be applied or redesigned for use against *Francisella*. Combination therapies targeting both LT and PBP enzymes are currently being evaluated for other pathogens, such as *P. aeruginosa*, and could prove to be effective against *F. tularensis* (Dik et al., 2019). Characterization of the PG enzymes in *Francisella* and their functions is an underdeveloped (but very promising) area of study. Research efforts in this field will likely uncover strategies to develop more effective antibiotics and inhibitors against tularemia.

## Author contributions

BB and JB contributed to the conceptualization, writing, and editing. All authors have read and agreed to the published version of the manuscript.

## Conflict of interest

The authors declare that the research was conducted in the absence of any commercial or financial relationships that could be construed as a potential conflict of interest.

## Publisher’s note

All claims expressed in this article are solely those of the authors and do not necessarily represent those of their affiliated organizations, or those of the publisher, the editors and the reviewers. Any product that may be evaluated in this article, or claim that may be made by its manufacturer, is not guaranteed or endorsed by the publisher.

## Author Disclaimer

Opinions, interpretations, conclusions, and recommendations are those of the authors and are not necessarily endorsed by the US Army.
